# Acute pontine infarction in a patient with 8-shaped basilar artery fenestration malformation: A case report

**DOI:** 10.1097/MD.0000000000029445

**Published:** 2022-07-08

**Authors:** WenSheng Zhang, WeiFang Xing, HongLi Gu, JinZhao He

**Affiliations:** a Department of Neurology, Heyuan People’s Hospital, Guangdong Provincial People’s Hospital Heyuan Hospital, Guangdong Province, China.

**Keywords:** arterial fenestration malformation, case report, local hemodynamic abnormalities, local thrombosis, transcranial Doppler

## Abstract

**Introduction:**

Cerebrovascular fenestration malformation is a relatively rare vascular dysplasia, and an 8-shaped basilar artery fenestration malformation is even rarer. The characteristics of transcranial Doppler cerebral blood flow in cerebrovascular fenestration malformations have rarely been studied or reported.

**Patient concerns:**

A 58-year-old woman presented with hypertension, diabetes, with no history of smoking or drinking. The patient had no relevant family history. The patient experienced left limb weakness for 2 days, which gradually worsened.

**Diagnosis:**

Head and neck computed tomography angiography revealed an 8-shaped fenestration deformity of the lower segment of the basilar artery with multiple stenoses of the local vessels. Transcranial Doppler cerebral blood flow examination at a depth of 85 cm revealed an eddy current in the lower segment of the basilar artery.

**Interventions:**

Tirofiban was administered intravenously for 3 days and subsequently changed to oral clopidogrel antiplatelet treatment.

**Outcomes:**

The modified Rankin Scale score at 3 months after disease onset was 0, indicating that the patient recovered well after treatment.

**Conclusion:**

A basilar artery 8-shaped fenestration is extremely rare and has seldom been reported. Cerebral vascular fenestration can lead to an acute cerebral infarction and its pathogenesis may include local hemodynamic abnormalities and thrombosis. Eddy currents can be detected by transcranial Doppler cerebral blood flow examination.

## 1. Introduction

Cerebrovascular fenestration malformation is a relatively rare vascular dysplasia^,[1]^ and an 8-shaped basilar artery fenestration malformation is even rarer.^[2]^ At present, the pathogenesis of cerebral infarction caused by cerebrovascular fenestration malformations has not been fully clarified^[3,4]^ and the characteristics of transcranial Doppler cerebral blood flow in cerebrovascular fenestration malformations have rarely been studied or reported. We report a case of acute pontine infarction in a patient with an 8-shaped fenestration deformity of the basilar artery and explore the characteristics of transcranial Doppler cerebral blood flow in a cerebrovascular fenestration malformation.

## 2. Case presentation

**Figure 1.**
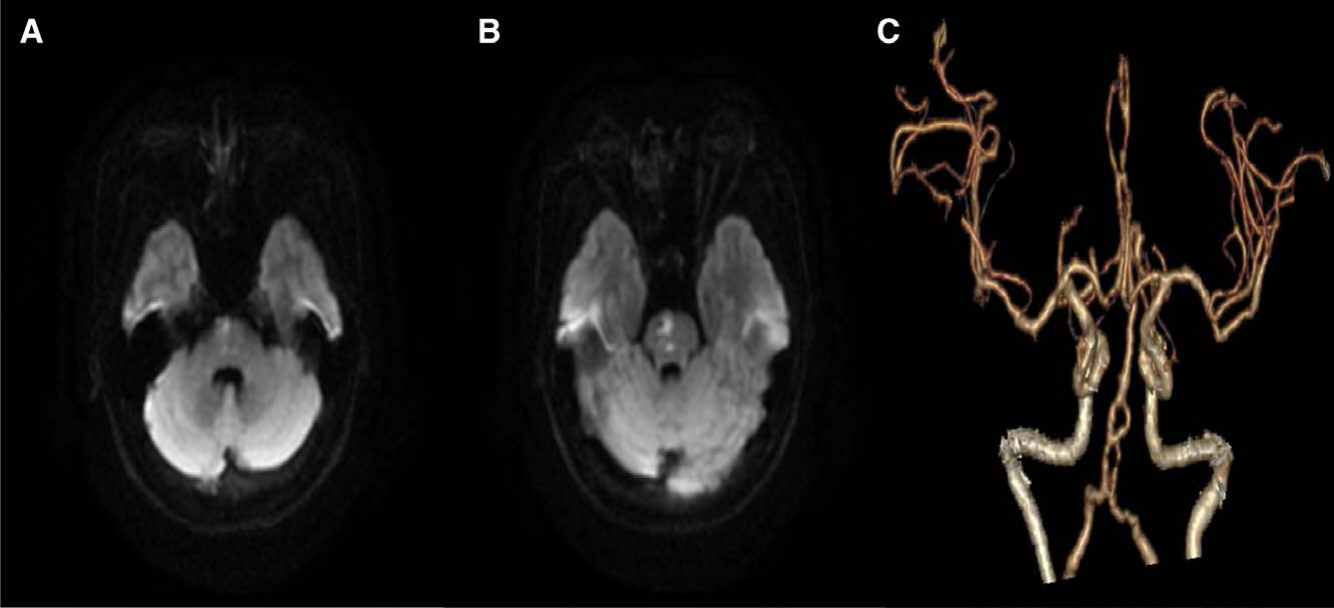
(A, B) Brain magnetic resonance imaging (MRI) findings, suggesting acute right pontine infarction. (C) Head and neck computed tomography angiography (CTA) showing an 8-shaped fenestration deformity in the lower segment of the basilar artery with multiple local stenoses.

**Figure 2.**
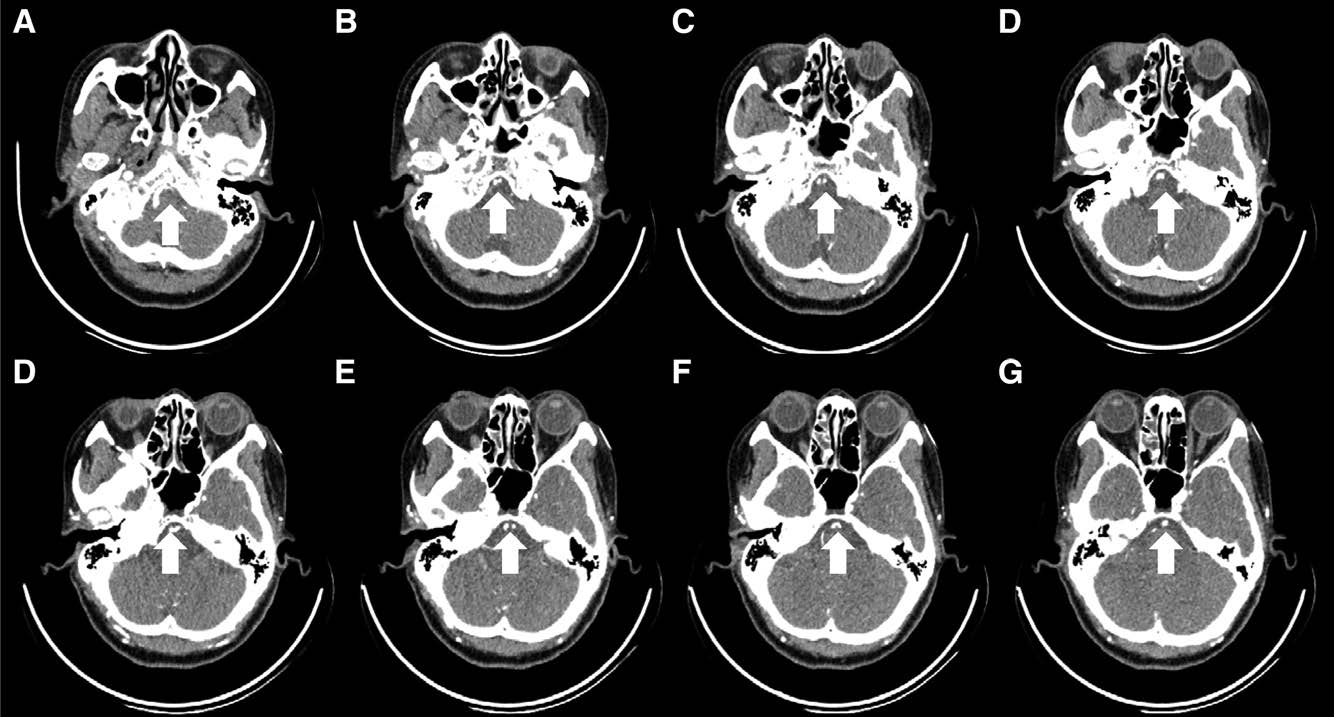
The original CTA images suggested an 8-shaped fenestration deformity in the lower segment of the basilar artery (as shown by the white arrow). The bilateral vertebral arteries merged at the intersection to reach the basilar artery (A). The basilar artery divided into 2 trunks in the lower segment and merged after traveling for some distance (B–D). After merging, the basilar artery divided into 2 trunks again, which in turn finally merged into a single trunk again (E–H). CTA = computed tomography angiography.

**Figure 3.**
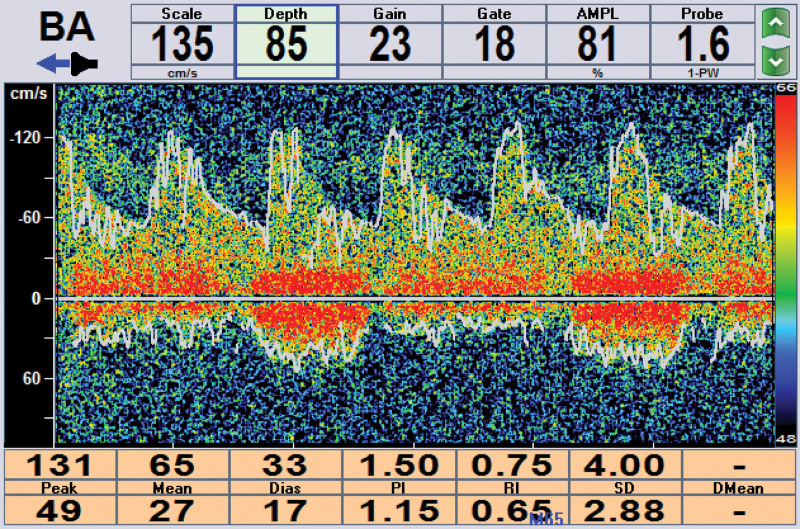
Transcranial Doppler cerebral blood flow examination at a depth of 85 cm revealed an eddy current in the lower segment of the basilar artery.

A 58-year-old woman presented with hypertension, diabetes, with no relevant family history and no smoking or drinking history. The patient experienced left limb weakness for 2 days, and the symptoms gradually worsened. The patient presented with unclear speech, left central facial paralysis, and grade 4 muscle strength of the left limb. The left finger-nose test and heel-knee-tibia test were slightly inaccurate. The National Institutes of Health Stroke Scale (NIHSS) score was 3 points. The brain computed tomography (CT) images showed no hemorrhage, while the brain magnetic resonance imaging (MRI) revealed a right pontine acute infarction (Fig. 1A and B). The head and neck CT angiography (CTA) records showed an 8-shaped fenestration deformity of the lower segment of the basilar artery with multiple stenoses of the local vessels (Fig. 1C). To rule out the possibility of aneurysm or dissection, we carefully reanalyzed the original CT angiography image in detail with the assistance of the imaging diagnostic physician. The bilateral vertebral arteries merged at the intersection where the basilar artery was formed (Fig. 2A), and then divided into 2 trunks in the lower segment and subsequently merged again after continuing for a certain distance (Fig. 2B–D). After the merge, the basilar artery further divided into 2 trunks before merging once more (Fig. 2E–H). Ultimately, an 8-shaped fenestration deformity was observed in the lower segment of the basilar artery. Although cerebral angiography is the gold standard for the diagnosis of cerebrovascular fenestration malformation, the patient and her family members refused to undergo the procedure. Transcranial Doppler cerebral blood flow examination at a depth of 85 cm revealed an eddy current in the lower segment of the basilar artery (Fig. 3).

The patient’s symptoms gradually worsened but did not rapidly reach a peak. Combined with the above-mentioned findings, the pathogenesis of the patient did not indicate a cardiogenic cerebral embolism. The patient’s right pontine infarction was located in the blood supply region that corresponded to the fenestration malformation of the basilar artery. Moreover, the anterior inferior cerebellar artery was considered to originate from the fenestration malformation. Therefore, the 8-shaped fenestration malformation of the lower segment of the basilar artery was considered the most likely cause of the right pontine acute infarction. Through transcranial Doppler cerebral blood flow examination at a depth of 85 cm, we observed an eddy current in the lower segment of the basilar artery. The pathogenesis of cerebral infarction may involve local hemodynamic abnormalities and local thrombosis. In terms of treatment, tirofiban was administered intravenously for 3 days and subsequently changed to oral clopidogrel antiplatelet treatment. After 1 week of treatment, the patient’s symptoms were completely relieved and she was discharged. The NIHSS score at discharge decreased to 0 points, indicating that treatment was effective. At follow-up, the patient had no new symptoms of acute cerebral infarction and the modified Rankin Scale (mRS) score at 3 months after onset was 0. Written informed consent was obtained from the patient for publication of this case report and its accompanying images.

## 3. Discussion

Cerebrovascular fenestration malformation refers to the limited repeated division of cerebral blood vessels into 2 branches before converging again after a certain distance.^[4]^ The incidence of cerebrovascular fenestration malformation is between 1.13% and 2.1%,^[5,6]^ among which basilar artery fenestration malformation is the most common.^[7]^

According to Wu et al,^[5]^ cerebrovascular fenestration malformations are classified according to shape into slit-like, convex-lens-like, duplication, and irregular types. In the present case, the basilar artery fenestration deformity was considered irregular. Stark et al^[2]^ reported a novel case of basilary artery double fenestration, which was complicated with an aneurysm and vertebral artery dissection. Our case of pontine infarction secondary to a basilar artery 8-shaped fenestration deformity is quite rare and has not been reported widely in the world. To the best of our knowledge, our case may be the first report of pontine infarction secondary to a basilar artery 8-shaped fenestration deformity in the world.

Several studies have reported cases of cerebrovascular fenestration associated with acute cerebral infarction and transient ischemic attack with a pathogenesis that may involve local hemodynamic abnormalities and local thrombosis.^[8,9]^ Transcranial Doppler cerebral blood flow examination at a depth of 85 cm of our patient revealed an eddy current in the lower segment of the basilar artery. We speculated that the specific pathogenesis of the acute pontine infarction may involve local hemodynamic abnormalities and local thrombosis. However, the specific pathogenesis of acute cerebral infarction caused by a fenestration malformation needs to be further explored by enrolling more cases.

In summary, basilar artery 8-shaped fenestrations have rarely been reported in the previous literature. Cerebral vascular fenestration can lead to an acute cerebral infarction and its pathogenesis may include local hemodynamic abnormalities and thrombosis. Eddy currents can be detected using transcranial Doppler cerebral blood flow examination.

## Author contributions

Data curation: W.Z., W.X.

Investigation: W.Z., H.G.

Methodology: W.Z., W.X.

Project administration: W.Z.

Supervision: J.H.

Writing – original draft: W.Z., W.X.

Writing – review & editing: W.Z., W.X.

## Acknowledgments

The authors thank Yangchun Wen and Guanghong Zhong for their assistance in preparing this manuscript.

## References

[1] Wang F Wang X Li X. A case of multiple vertebrobasilar artery fenestration misdiagnosed as vertebral artery dissection. BMC Neurol. 2020;20:63 32079531 10.1186/s12883-020-01642-2 PMC7033924

[2] Stark MM Skeik N Delgado Almandoz JE. Concurrent basilar artery double fenestration with aneurysm and vertebral artery dissection: case report and literature review of rare cerebrovascular abnormalities. Ann Vasc Surg. 2013;27:497.e15–21. 10.1016/j.avsg.2012.06.017 23548267

[3] Yamaguchi S Horie N Tsunoda K. Bow hunter’s stroke due to stretching of the vertebral artery fenestration: a case report. NMC Case Rep J. 2014;2:9–11. 28663954 10.2176/nmccrj.2014-0075 PMC5364926

[4] Jeong SK Kwak HS Cho YI. Middle cerebral artery fenestration in patients with cerebral ischemia. J Neurol Sci. 2008;275:181–4. 18801495 10.1016/j.jns.2008.07.037

[5] Wu X Chen X Zhu J. Imaging detection of cerebral artery fenestrations and their clinical correlation with cerebrovascular diseases. Clin Imaging. 2020;62:57–62. 32066034 10.1016/j.clinimag.2020.01.012

[6] Cooke DL Stout CE Kim WT. Cerebral arterial fenestrations. Interv Neuroradiol. 2014;20:261–74. 24976087 10.15274/INR-2014-10027 PMC4178766

[7] Woo SR Seo MW Kim YH. Extreme duplication-type, nonseparated fenestration of the basilar artery in a patient with pontine infarction: confirmation with virtual arterial endoscopy. J Clin Neurol. 2006;2:74–7. 20396489 10.3988/jcn.2006.2.1.74 PMC2854947

[8] Palazzo P Ruff M Lyerly MJ. Basilar artery thrombus vs. fenestration: a differential diagnostic challenge in acute ischemic stroke. J Neuroimaging. 2014;24:607–9. 24251913 10.1111/jon.12069

[9] Gold JJ Crawford JR. An unusual cause of pediatric stroke secondary to congenital basilar artery fenestration. Case Rep Crit Care. 2013;2013:627972. 24804123 10.1155/2013/627972 PMC4010040

